# Clinical validation of an optimized multimodal neurocognitive assessment of chronic mild TBI

**DOI:** 10.1002/acn3.51020

**Published:** 2020-03-23

**Authors:** Mark L. Ettenhofer, Sarah I. Gimbel, Evelyn Cordero

**Affiliations:** ^1^ Uniformed Services University of the Health Sciences Bethesda Maryland; ^2^ Defense and Veterans Brain Injury Center Silver Spring Maryland; ^3^ Naval Medical Center San Diego San Diego California; ^4^ University of California San Diego California; ^5^ General Dynamics Information Technology Falls Church Virginia; ^6^ Henry M. Jackson Foundation Bethesda Maryland

## Abstract

**Objective:**

Previous laboratory‐based studies have shown that neurocognitive eye‐tracking metrics are sensitive to chronic effects of mild traumatic brain injury (mTBI), even in individuals with normal performance on traditional neuropsychological measures. In this study, we sought to replicate and extend these findings in a military medical environment. We expected that metrics from the multimodal Fusion n‐Back test would successfully distinguish chronic mTBI participants from controls, particularly eye movement metrics from the more cognitively challenging “1‐Back” subtest.

**Methods:**

We compared performance of participants with chronic mTBI (*n* = 46) and controls (*n* = 33) on the Fusion n‐Back test and a battery of conventional neuropsychological tests. Additionally, we examined test reliability and the impact of potential confounds to neurocognitive assessment.

**Results:**

Our results supported hypotheses; Fusion 1‐Back metrics were successful in multimodal (saccadic and manual) classification of chronic mTBI versus control. In contrast, conventional neuropsychological measures could not distinguish these groups. Additional findings demonstrated the reliability of Fusion n‐Back test metrics and provided evidence that saccadic metrics are resistant to confounding influences of age, intelligence, and psychiatric symptoms.

**Interpretation:**

The Fusion n‐Back test could provide advantages in differential diagnosis for complex brain injury populations. Additionally, the rapid administration of this test could be valuable for screening patients in clinical settings where longer test batteries are not feasible.

## Introduction

Mild traumatic brain injury (mTBI) has a worldwide incidence of approximately 224/100,000 individuals.^1^ Incidence of mTBI is even higher within military populations due to demographic characteristics and physical hazards associated with military operational/training activities.^2^ Since 2000, U.S. military personnel have sustained over 340,000 mTBIs in both combat and garrison environments;^3^ for up to 20%, post–concussive symptoms such as irritability, fatigue, or difficulty concentrating may persist into the chronic phase of injury (beyond 3 months), disrupting life activities and motivating patients to seek follow‐up care.^4^, ^5^, ^6^, ^7^ These symptoms, however, can be influenced by a wide range of neurological and nonneurological factors.^8^, ^9^, ^10^

Comprehensive neuropsychological evaluation is a widely accepted approach for identification of functional neural impairment;^11^ however, this approach generally returns “normal” results beyond 90 days postinjury.^12^, ^13^, ^14^, ^15^ This raises questions about the value of neuropsychological assessment for the mTBI patient who seeks treatment after symptoms have become chronic. Accurate and efficient identification of chronic neural impairment related to mTBI is critical to guide treatments to reduce post–concussive symptoms and return the individual to optimum functioning.

Measures of oculomotor performance show considerable potential to improve assessment of mTBI. Several studies have shown that mTBI has a negative impact on eye movements,^14^, ^15^, ^16^, ^17^, ^18^, ^19^ particularly with multiple injuries or persistent post–concussive symptoms.^14^, ^20^, ^21^ Eye movement abnormalities in TBI could be related to the broad network of regions that must communicate effectively to acquire and integrate information from the visual environment.^22^ Impaired eye movements have been linked to axonal injury in postmortem tissue and on diffusion tensor imaging;^23^, ^24^, ^25^ abnormal functional connectivity in chronic mTBI extends to the visual system and its interactions with higher‐order cognitive processing.^26^ Importantly, the types of eye movements that are most strongly impacted by mTBI are those that most heavily rely upon effective cognitive processing, as opposed to measures of basic neuromotor function.^14^, ^18^, ^27^, ^28^, ^29^ In particular, previous research conducted by our group ^14^, ^15^ and others ^28^, ^29^, ^30^ has shown enhanced sensitivity of eye movements to effects of mTBI under conditions of increased cognitive load. Many oculomotor metrics are also less impacted by age, education, or intelligence, relative to conventional neuropsychological measures.^20^, ^31^, ^32^

Concurrent measurement of multiple response modalities while an examinee completes a cognitive test may provide a means for improved assessment of chronic mTBI.^31^ In previous studies,^14^, ^15^, ^31^, ^33^, ^34^ our group has developed and validated methods to assess saccadic eye movements and manual motor performance in response to varying levels of cognitive load. This study extends this line of research using a more advanced system optimized to be clinically feasible for assessment of TBI in real‐world medical environments. Based on recent findings, we hypothesized that the Fusion n‐Back test^15^ – particularly, saccadic metrics derived from the more cognitively challenging 1‐Back subtest – would outperform conventional neuropsychological measures in distinguishing participants with chronic mTBI from controls.

## Methods

### Participants

A volunteer sample of U.S. Active Duty military personnel and Veterans was recruited from military treatment facilities in the San Diego area. The mTBI group consisted of adults (>18 years old) with persistent symptoms related to mTBI^35^ sustained 3 months to 12 years previously; the control group had no history of TBI or other neurological conditions. Of 120 participants enrolled, *n* = 79 (*n* = 33 control; *n* = 46 mTBI) met full eligibility requirements and were included in analyses. Participants with moderate‐to‐severe TBI (*n* = 15), TBI that fell outside the allotted time window (*n* = 11), medical conditions other than TBI that would be expected to impact performance (*n* = 7), incomplete testing session and loss to follow‐up (*n* = 2), and failure on two or more measures of response validity/effort or noncompliance with task instructions (*n* = 6) were excluded from analysis.

### Procedures

After providing written informed consent, participants provided demographic information and medical history (Table 1). TBI history was obtained using the Ohio State University TBI Identification Method (OSU TBI‐ID^36^, ^37^) and confirmed using medical records. Participants then completed a fixed battery of standardized self‐report and neuropsychological measures and the Fusion n‐Back test, described below. This study was approved by the Institutional Review Board at Naval Medical Center San Diego. Neuropsychological tests are detailed in Table 2 *.* ^38^, ^39^, ^40^, ^41^, ^42^, ^43^, ^44^, ^45^, ^46^, ^47^

**Table 1:** Participant characteristics.

	Control	Mild TBI	*P* ^1^
*N*	33	46	–
Female, *n* (%)	12 (36.4%)	16 (34.8%)	0.89
Age in years, M (SD)	28.91 (6.45)	32.09 (7.55)	0.05
Education in years, M (SD)	14.97 (2.62)	14.28 (2.01)	0.19
U.S. Military Duty Status			0.14
Active duty	33 (100.0%)	48 (94.1%)	
Retired/Separated	0 (0.0%)	3 (5.9%)	
U.S. military branch of service			0.61
Army *n* (%)	1 (3.0%)	3 (6.5%)	
Navy *n* (%)	31 (93.9%)	39 (84.8%)	
Marine Corps *n* (%)	1 (3.0%)	3 (6.5%)	
Air Force	0 (0.0%)	1 (2.2%)	
Race/Ethnicity			0.70
Hispanic or Latino *n* (%)	6 (18.2%)	12 (26.1%)	
Black/African American, *n* (%)	1 (3.0%)	2 (4.3%)	
White/Caucasian, *n* (%)	22 (66.7%)	28 (60.9%)	
Asian/Pacific Islander, *n* (%)	1 (3.0%)	1 (2.2%)	
Other, *n* (%)	3 (9.1%)	3 (6.5%)	
No. of Lifetime Mild TBIs, Md (IQR)	–	3 (2, 5)	
Cause of Most Recent Injury			–
Motor Vehicle Accident	–	9 (19.6%)	
Sports/Recreation	–	13 (28.3%)	
Fall/Accident	–	12 (26.1%)	
Assault/Combat	–	6 (13.0%)	
Military Training	–	6 (13.0%)	
NSI‐22 total score, M (SD)	7.85 (10.86)	27.07 (14.97)	<0.001
PHQ‐8 total score, M (SD)	3.09 (4.56)	9.54 (5.80)	<0.001
PCL‐5 total score, M (SD)	8.88 (13.64)	26.22 (15.85)	<0.001

^1^ Statistical significance of independent samples t‐test or chi‐square, as appropriate.

**Table 2:** Test performance in control and chronic mild TBI groups.

		Control			Chronic Mild TBI					
		M	SD	% Impaired^1^	M	SD	% Impaired^1^	*d* ^2^	AUC	Exp(B)^3^ [95% CI]
Neuropsychological Tests										
Estimated Premorbid IQ (SS)		108.52	11.40	–	106.91	11.30	–	−0.14	–	–
Global Cognition (*t*)		53.61	6.37	0.0%	53.58	7.69	2.2%	0.00	–	–
Trail Making Test A (*t*)		55.12	13.35	15.2%	55.57	11.82	6.5%	0.04	–	–
Trail Making Test B (*t*)		54.61	11.09	9.1%	51.11	11.46	17.4%	−0.31	–	–
Digit Span Forward (SS)		10.03	2.54	15.2%	10.63	3.28	19.6%	0.21	–	–
Digit Span Backward (SS)		10.27	3.39	9.1%	10.96	3.14	8.7%	0.21	–	–
Digit Span Sequencing (SS)		11.97	2.86	3.0%	12.07	2.97	2.2%	0.03	–	–
Symbol Search (SS)		11.30	2.89	9.1%	10.78	3.00	8.7%	−0.18	–	–
Fusion n‐Back Test										
0‐Back	Saccadic RT Latency (ms)	316.10	87.64	9.1%	337.23	92.34	21.7%	0.23	–	–
	Saccadic RT Variability (ms)	119.73	52.31	12.1%	145.86	60.76	32.6%	0.46	–	–
	Saccadic Inhibition Errors (%)	10.29	11.84	12.1%	16.12	16.67	21.7%	0.41	–	–
	Manual RT Latency (ms)	674.30	137.05	18.1%	701.08	163.98	23.9%	0.18	–	–
	Manual RT Variability (ms)	137.46	41.56	12.1%	135.36	40.85	10.7%	−0.05	–	–
	Color Discrimination Score	84.01	3.98	12.1%	81.85	12.34	15.2%	−0.27	–	–
1‐Back	Saccadic RT Latency (ms)	293.56	69.40	12.1%	322.20	92.31	26.1%	0.35	–	–
	Saccadic RT Variability (ms)	131.87	56.85	18.2%	160.77	66.74	34.8%	0.47^†^	0.63*	1.54 [.97‐2.11]
	Saccadic Inhibition Errors (%)	6.56	10.60	15.2%	14.33	11.84	32.6%	0.69**	0.75***	1.81 [1.12‐2.54]
	Manual RT Latency (ms)	775.74	117.19	15.2%	862.19	123.54	30.4%	0.72*	0.70**	–
	Manual RT Variability (ms)	197.22	32.93	15.2%	201.70	37.67	17.4%	0.13	–	–
	Working Memory Score	59.33	7.29	12.1%	47.73	16.93	56.5%	−0.96**	0.74***	2.30 [1.39‐3.26]

M, mean; SD, standard deviation; t, age‐corrected *t* score; SS, age‐corrected scaled score.

^1^ Percentage of participants performing ≥ 1SD more poorly than the control group (Fusion n‐Back) or normative (neuropsychological test) mean.

^2^ Statistical significance provided for ANCOVA covarying age: *P < 0.05, **P < 0.01, ***P < 0.001, †*P* < 0.10.

^3^ Odds ratios for mild TBI group membership associated with 1SD poorer performance relative to the control group on each predictor (from accepted logistic regression model).

### Fusion n‐Back test

The Fusion n‐Back test^15^ is a multimodal cognitive task that combines the working memory demands of the classic ‘n‐Back’ task^48^ with the eye movement and visual attention demands of the Bethesda Eye & Attention Measure (BEAM) task.^31^ In preparation for this study, development efforts were undertaken to optimize the previously described^15^ Fusion prototype for enhanced mobility, efficiency, and clinical feasibility. In this study, the Fusion n‐Back test was administered on a laptop computer, and testing procedures were streamlined, reducing testing time to 12 min total, plus instructions. Additionally, testing software was upgraded to provide enhanced ease‐of‐use, automated corrective feedback if an examinee failed to follow task instructions, and fully automated data processing/scoring.

**Figure 1:**
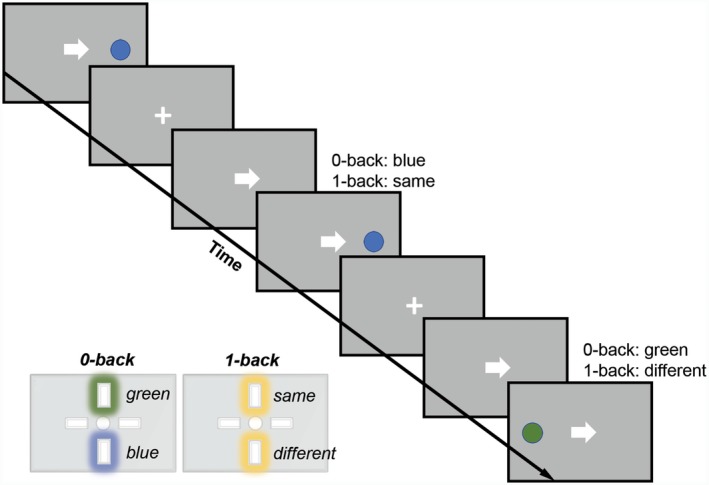
Fusion n‐Back Test Stimuli. Target circles were green or blue, presented either to the left or right of the central fixation cross. Participants were instructed for each test trial to look at the target and press the correct button as quickly as possible. Trials were presented in a pseudorandom order, with 56 directional cues and 32 misdirectional cues, starting with 10 practice trials per cognitive load condition. The test was performed across multiple conditions: Low Load (0‐Back/Color Discrimination; press the button representing green vs. blue current target), and High Load (1‐Back/Working Memory; press the button representing same vs. different color target relative to the previous target).

The Fusion n‐Back test measures saccadic and manual responses to visual targets across two levels of cognitive load (0‐Back and 1‐Back; see Fig. 1). Details of the test have been described previously;^15^ primary test metrics are shown in Table 3. Eye‐tracking data were acquired at 150Hz using a Gazepoint GP3 HD Eye Tracker. Calibration was performed at the beginning of each task run using a 9‐point rectangular calibration screen. Manual responses were recorded with a Cedrus RB‐530 response pad. Stimuli were presented using PsychoPy v1.85.3 at 1920x1080 resolution on a 15” 60Hz LCD notebook computer display. Participants were seated with eyes positioned 24” from the stimulus display. Head movements were minimized with a chin rest. Gaze data and manual responses were synchronized with task event markers during data acquisition.

**Table 3:** Psychometric characteristics of fusion n‐Back test metrics.

		Reliability^1^	Age (r^3^)	Education (r^4^)	IQ (r^4^)	Global Cognition (r^4^)	PCL‐5 (r^4^)	PHQ‐8 (r^4^)
0‐Back	Saccadic RT Latency (ms)	0.94	0.10	−0.06	−0.06	−0.14	0.13	0.14
	Saccadic RT Variability (ms)	0.76	0.00	−0.02	−0.18	−0.16	0.05	0.08
	Saccadic Inhibition Errors (%)	0.84	0.05	−0.12	−0.09	−0.17	−0.08	−0.14
	Manual RT Latency (ms)	0.99	0.06	−0.03	−0.23*	−0.37**	0.11	0.19
	Manual RT Variability (ms)	0.86	0.02	−0.11	−0.25*	−0.32**	0.12	0.13
	Color Discrimination Score	0.99	−0.06	−0.02	−0.02	0.35**	−0.19^†^	−0.17
1‐Back	Saccadic RT Latency (ms)	0.95	0.09	0.03	0.09	−0.15	0.06	0.05
	Saccadic RT Variability (ms)	0.77	0.11	0.01	−0.03	−0.02	−0.04	0.01
	Saccadic Inhibition Errors (%)	0.76	−0.18	−0.09	−0.04	0.00	−0.10	−0.18
	Manual RT Latency (ms)	0.95	0.07	0.02	−0.22^†^	−0.42***	0.08	0.14
	Manual RT Variability (ms)	0.70	0.25*	−0.00	−0.13	−0.29*	0.15	0.14
	Working Memory Score	0.96	0.12	0.04	0.18	0.28**	−0.06	−0.02
Global Cognition		0.75^2^	–	0.14	0.27*	–	−0.02	−0.09

Note

IQ = estimated premorbid intelligence. Global cognition, as derived from age‐corrected neuropsychological tests, is included for comparison to Fusion n‐Back test metrics.

^1^ Split‐half correlation with Spearman‐Brown correction, except where otherwise indicated.

^2^ Cronbach’s alpha derived from age‐corrected standardized test scores.

^3^ Partial correlation controlling for group (chronic mild TBI vs. control).

^4^ Partial correlation controlling for age and group.

* *P* < 0.05.

** *P <* 0.01.

*** *P* < 0.001.

^†^ *P* < 0.10.

### Statistical analyses

Gaze data from the Fusion n‐Back test were processed using custom software, which automatically removed invalid trials and coded saccadic and manual responses. Standardized t‐scores from the conventional neuropsychological battery were averaged to represent ‘global cognition,’ with better performance yielding higher scores. Statistical analyses were conducted using SPSS 25.0. A two‐tailed alpha level of .05 was used for all analyses. Missing data (2.8% of all primary metrics) were imputed using expectation maximization. Chi‐square analyses and independent‐samples t‐tests were used to compare demographic characteristics and self‐reported symptoms between groups.

Analyses were conducted to compare Fusion n‐Back test performance between groups and to identify a robust set of variables for use in identification of chronic mTBI. Mean test performance was compared between groups using ANOVA (for age‐corrected neuropsychological tests) or ANCOVA controlling for age (for Fusion metrics). Receiver operating characteristic (ROC) analyses were conducted to identify Fusion n‐Back metrics with the greatest sensitivity/specificity for classifying chronic mTBI versus control groups. ROC analysis predictors were selected based on the subset of variables that were *P *< 0.10 in ANOVA/ANCOVA. Forward stepwise logistic regression models were then used to evaluate joint classification accuracy of multiple metrics, with predictors selected based on the subset of variables that were *P *< 0.10 in ROC analyses, plus age. For these regressions, *p*‐values were generated by bootstrapping across 1000 samples.

Reliability, sensitivity, and specificity to effects of chronic mTBI, correspondence with global cognitive performance, and estimates of effects of common confounds and psychiatric comorbidities were examined to evaluate potential use of this technology for assessment of mTBI in future research and clinical settings. Reliability analyses were conducted using split‐half correlation with Spearman‐Brown adjustment (for Fusion n‐Back metrics) or Cronbach’s alpha (for global cognition, based upon individual standardized scores from the battery of conventional neuropsychological tests). Partial correlations controlling for age and group (chronic mTBI vs. control) were used to evaluate relationships of Fusion metrics with demographic characteristics, psychiatric symptoms, and global cognition.

## Results

### Participant characteristics

Participant characteristics are presented in Table 1. Mild TBI (*n* = 46) and control (*n* = 33) groups did not differ on demographic or military variables. However, there was a trend for older age in the mTBI group (M = 32.09, SD = 7.55) relative to the control group (M = 28.91, SD = 6.45), *P *= .054. Consistent with inclusion criteria, the mTBI group demonstrated elevated neurobehavioral symptoms on the NSI relative to the control group. The mTBI group also demonstrated elevated levels of depression (PHQ‐8) and post‐traumatic stress (PCL‐5) relative to the control group.

### Comparison of neurocognitive performance in mTBI versus control groups

Test performance by group is shown in Table 2. Groups did not differ on premorbid IQ, global cognition, or any individual neuropsychological performance metrics examined. However, on the Fusion 1‐Back subtest, mean performance of the mTBI group was poorer than that of the control group on saccadic inhibition errors (*d *= .69, *P *< 0.01), manual RT latency (*d *= .72, *P *< 0.05), and working memory score (*d *= −0.96, *P *< 0.01). There was also a trend for greater saccadic RT variability in the mTBI group on the 1‐Back subtest (*d *= 0.47, *P *= 0.07).

**Figure 2:**
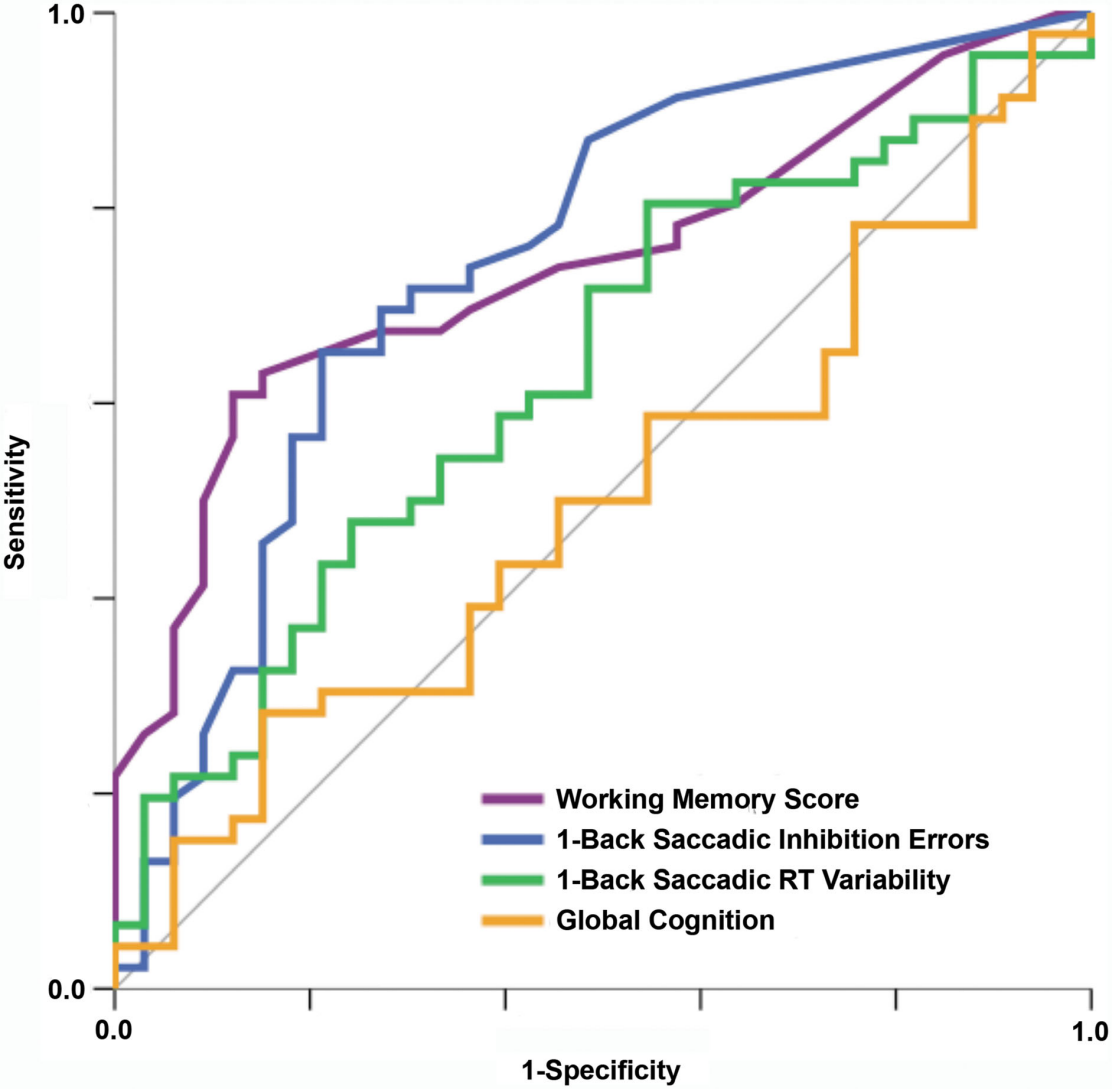
ROC Curve for Classification of Chronic Mild TBI versus Control Group. Global cognition shown for reference; all other metrics shown represent predictors (all *P* < 0.05) in the final logistic regression model, χ^2^(3) = 25.90,* P* < 0.001. The model explained 38% (Nagelkerke *R^2^*) of the variance in group (chronic mild TBI vs. control) and correctly classified 74.7% of cases, with positive predictive value (PPV) of 0.76 and negative predictive value (NPV) of 0.72.

**Figure 3:**
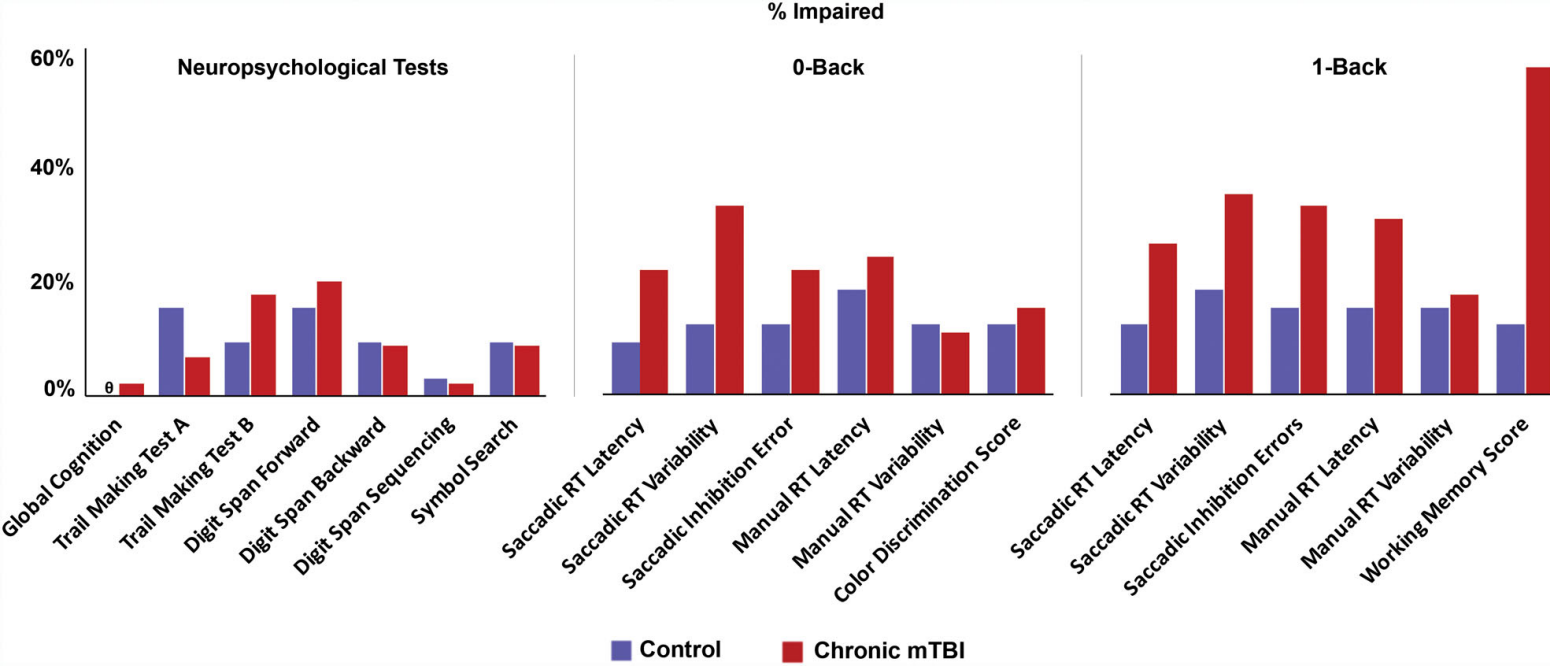
Comparison of % Impaired in Mild TBI versus Control Group. Metrics shown represent neuropsychological tests (left) and saccadic and manual metrics for the Fusion 0‐Back (low load, middle) and Fusion 1‐Back (high load, right) tests for the control group (purple) and chronic mild TBI group (red). X‐axis represents % identified as impaired in each measure. “Impaired” is greater than or equal to 1 standard deviation poorer than mean of the control group. θ Global Cognition in the control group had a value of 0.

Next, analyses were performed to identify a robust set of Fusion n‐Back metrics for identification of chronic mTBI. ROC analyses were conducted for the subset of variables that were *P *< 0.10 in ANCOVAs. As shown in Table 2, these analyses provided similar results to ANCOVA, with the addition of saccadic RT variability on the 1‐Back subtest (AUC = 0.63, *P* < 0.05) as a significant classifier of chronic mTBI versus control group. AUC values for this group of individual metrics ranged from 0.63 to 0.75. Logistic regression was then performed to examine combined/incremental value of Fusion metrics for identification of chronic mTBI. All variables with significant AUC values from ROC analysis were entered as standardized z‐scores using a stepwise forward method with *P *< 0.10 for entry. Regression diagnostics demonstrated that multicollinearity was not present for any variables (variance inflation factor < 4), and the model was well calibrated (Hosmer‐Lemeshow test *P *> 0.20 for all steps). Model improvements for steps 1‐3 were significant, *P *< 0.05. The accepted model (step 3) explained 38% (Nagelkerke *R* ^2^) of the variance in group (chronic mTBI vs. control), χ^2^(3) = 25.90,* P *< 0.001, correctly classifying 74.7% of cases. In an additional step, age was entered as a predictor, but it did not significantly improve model fit (*P *> 0.05); therefore, this model was rejected. Effect sizes of individual predictors were comparable between model steps 3 and 4. ROC curves for variables in the accepted logistic regression model are presented in Figure 2. Proportions of participants impaired in each group are presented for each metric in Figure 3.

Odds ratios for variables included in the accepted logistic regression model are presented in Table 2; all successful predictors in this model were derived from the Fusion 1‐Back subtest. As shown, participants with poor working memory scores (*z* = −1.0) were 2.30x more likely to be in the chronic mTBI group, Wald = 8.11, *P *= 0.001 (95% CI = 1.39–3.26). Membership in the mTBI group was 1.81x more likely (95% CI = 1.12–2.54) among participants with elevated saccadic inhibition errors (*z* ≥ 1.0; Wald = 5.38, *P *= 0.04) and 1.54x more likely (95% CI = 0.97–2.1) among participants with elevated saccadic RT variability (*z* ≥ 1.0; Wald = 3.43, *P *= 0.03). Combined value of these metrics was high, with positive predictive value of .76 and negative predictive value of .72.

### Psychometric characteristics of the Fusion n‐Back test

Additional psychometric characteristics of Fusion n‐Back metrics are presented in Table 3. Overall, reliability estimates for Fusion n‐Back metrics ranged from acceptable to excellent. Split‐half reliability ranged from *r*_sb_ = 0.76 to *r*_sb_ = 0.95 for saccadic metrics and *r*_sb_ = 0.70 to *r*_sb_ = 0.99 for manual metrics. As a point of comparison, Cronbach’s alpha was 0.75 for global cognition.

Partial correlations were used to evaluate relationships of Fusion metrics with age (controlling for group), as well as education, IQ, global cognition, and self‐reported symptoms of post‐traumatic stress and depression (each controlling for age and group). Increased age was associated with greater manual RT variability on the 1‐Back subtest, *r*_p_=0.25, *P* < 0.05, however, neither age nor education were significantly associated with any other Fusion n‐Back test metrics. Higher estimated premorbid IQ was associated with faster manual RT latency (*r*_p_ = −0.23, *P *< 0.05) and reduced manual RT variability (*r*_p_ = −0.25, *P *< 0.05) on the 0‐Back subtest. Higher estimated premorbid IQ was also associated with higher levels of global cognition, r_p_ = 0.27, *P *< 0.05.

Higher levels of global cognition were associated with better performance on all manual metrics from the Fusion n‐Back test, including manual RT latency (0‐Back: *r*_p_ = −0.37, *P *< .01; 1‐Back: *r*_p_ = −0.42, *P *< 0.001), manual RT variability (0‐Back: *r*_p_ = −0.32, *P *< 0.01; 1‐Back: *r*_p_ = −0.29, *P *< 0.05), and manual response accuracy (0‐Back/Color Discrimination: *r*_p_ = 0.35, *P *< 0.01; 1‐Back/Working Memory: *r*_p_ = 0.28, *P *< 0.01). Saccadic metrics were not associated with conventional measures of global cognition.

## Discussion

This study was designed to evaluate an optimized version of the Fusion n‐Back test^15^ for multimodal neurocognitive assessment of chronic mTBI. Consistent with previous findings obtained in laboratory settings,^14^, ^15^ the Fusion n‐Back test also demonstrated good sensitivity and specificity for chronic mTBI when used with U.S. military personnel in a clinical setting. As shown previously,^14^, ^15^ in this study, conventional neuropsychological tests again failed to discriminate between individuals with chronic mTBI and controls. Additional psychometric strengths of the Fusion n‐Back Test included high levels of internal reliability and – for the best‐discriminating metrics – no detectable confounding by demographic or psychiatric factors. Overall, these findings support the use of neurocognitive eye tracking for clinical assessment of individuals with chronic mTBI. As expected,^15^ the more‐challenging 1‐Back subtest provided superior classification of chronic mTBI relative to the 0‐Back subtest. Considering similar findings with other eye movement tasks,^28^, ^29^, ^30^ it appears likely that the additional working memory demands were instrumental in illuminating impairment related to chronic mTBI.

Additionally, current findings provide clear evidence for the value of a multimodal approach to neurocognitive assessment. By measuring concurrent eye movements and manual responses to test stimuli, the Fusion n‐Back test produces distinct sets of saccadic and manual metrics. Examined individually, two of three saccadic metrics and two of three manual metrics from the Fusion 1‐Back subtest were demonstrably poorer within the chronic mTBI group. When this set of variables was evaluated together, a set of three best‐performing Fusion n‐Back metrics (two saccadic, one manual) emerged as complementary predictors of TBI group.

The value of assessing multiple neurocognitive and motor processes appears to be heightened by the heterogeneous nature of neural dysfunction experienced by individuals with chronic mTBI. Approximately 30–50% of individual chronic mTBI group participants demonstrated impairments in each of the best‐performing metrics, including saccadic RT variability, inhibition errors, and working memory score. Collectively, these metrics were better able to identify chronic mTBI than any one test metric alone. While none of the individual test metrics was a diagnostic “silver bullet,” the Fusion 1‐Back subtest appeared to effectively tap into multiple common forms of neural impairment associated with effects of chronic mTBI.

Aside from identifying persistent effects of neural injury, assessment of cognitive strengths and weaknesses can provide valuable information about an individual’s capacity to complete real‐world functional tasks.^49^, ^50^, ^51^ In this study, manual metrics from the Fusion n‐Back test were consistently and robustly associated with global cognitive performance. Therefore, these manual metrics may also be useful in predicting functional impairment, as defined by conventional neuropsychological measures with established (if modest) predictive relationships with functional capacity.^49^ The functional relevance of the types of saccadic impairment elicited by the Fusion n‐Back test has not yet been examined directly. However, the inconsistent and disinhibited eye movements demonstrated by many chronic mTBI participants in this study could reduce real‐world performance by interfering with the acquisition of visual information from the environment. Even among individuals who are functionally intact, these saccadic impairments might serve as valuable biomarkers of neuronal injury. Additional research will be needed to investigate the functional relevance of different forms of saccadic impairment in comparison to conventional cognitive measures.

We also observed a psychometric divergence between saccadic and manual metrics in their relationships with estimated premorbid IQ and psychiatric symptoms. Consistent with previous research,^20^, ^31^, ^32^ estimated intelligence was related to conventional neuropsychological measures and multiple manual metrics, but was not related to any saccadic metrics. Similarly, symptoms of depression and posttraumatic stress were related to manual – but not saccadic – performance. These findings provide additional evidence that saccadic metrics may provide advantages over manual metrics for detection of chronic mTBI effects with minimal interference from demographic or psychiatric factors that can confound conventional measures of cognitive performance. Interestingly, performance on the Fusion 1‐Back subtest was sensitive to chronic mTBI, while the ostensibly more challenging Digit Span test from the conventional neuropsychological battery was not. The heightened sensitivity of Fusion n‐Back metrics to chronic mTBI may be related to a synergy of multiple cognitive and motor demands embedded within this multimodal task.

While the mTBI and control groups were generally well matched, inclusion criteria restricted the mTBI group to those participants reporting persistent post–concussive symptoms. This criterion was selected to maximize clinical relevance of findings, as individuals are unlikely to seek clinical care if they feel their symptoms have resolved. As expected based on patterns of comorbidity, this symptomatic group of patients also had higher levels of depression and posttraumatic stress than the control group. However, these psychiatric symptoms were not related to primary metrics from the Fusion n‐Back test, so we opted not to control for these factors in our analyses. Additional research within a larger sample may be useful to evaluate potentially subtle effects of psychiatric status on saccadic versus manual test metrics.

This study, building upon previous iterations of the Fusion system,^14^, ^15^, ^31^, ^33^, ^34^ provides compelling support for the utility of the multimodal neurocognitive assessment of chronic mTBI. Consistent with our previous findings,^14^, ^15^ the clinically optimized version of the Fusion n‐Back test used in this study successfully discriminated between service members with chronic mTBI and a well‐matched group of controls. Fusion metrics were most sensitive under the higher cognitive load condition (1‐Back subtest). In contrast, conventional neuropsychological measures were unable to distinguish chronic mTBI and control groups. Additional findings supported the reliability of the Fusion test and suggested that saccadic metrics may be uniquely resistant to confounding influences of age, intelligence, and psychiatric symptoms. These test characteristics could provide advantages in differential diagnosis for complex brain injury populations. Additionally, with testing time as short as 8 min (if the 1‐Back subtest is used alone), the Fusion system could be valuable for screening patients within clinical settings where longer test batteries are infeasible. Follow‐up research is needed to identify changes in multimodal test performance over time, including comparisons of pre‐ and postinjury measurements and examination of potential improvement across the acute and subacute stages of TBI recovery.

## Conflict of Interest

The technology described in this manuscript is included in U.S. Patent Application No. 61/779,801, U.S. Patent Application No. 14/773,987, European Patent Application No. 14780396.9, and International Patent Application No. PCT/US2014/022468, with rights assigned to the Uniformed Services University of the Health Sciences. Dr. Ettenhofer is a named inventor on these patents. All authors report no competing financial interests exist.

## Author Contribution

Ettenhofer contributed to the conception and design of the study; Ettenhofer, Gimbel, and Cordero contributed to the acquisition and analysis of data; Ettenhofer and Gimbel wrote the manuscript and prepared the figures, with contributions from Cordero.
